# Biological effects of exposure to static electric fields in humans and vertebrates: a systematic review

**DOI:** 10.1186/s12940-017-0248-y

**Published:** 2017-04-17

**Authors:** Anne-Kathrin Petri, Kristina Schmiedchen, Dominik Stunder, Dagmar Dechent, Thomas Kraus, William H. Bailey, Sarah Driessen

**Affiliations:** 1 Research Center for Bioelectromagnetic Interaction, RWTH Aachen University, Pauwelsstraße 30, 52074 Aachen, Germany; 2 Center for Occupational and Environmental Health Risk Assessment, Exponent, 17000 Science Drive, Suite 200, Bowie, MD 20715 USA

**Keywords:** Static electric fields, High-voltage direct current, Power line, Exposure, Field perception, Physiological functions, Biological effects

## Abstract

**Background:**

High-voltage direct current (HVDC) lines are the technology of choice for the transport of large amounts of energy over long distances. The operation of these lines produces static electric fields (EF), but the data reviewed in previous assessments were not sufficient to assess the need for any environmental limit. The aim of this systematic review was to update the current state of research and to evaluate biological effects of static EF.

**Methods:**

Using the PRISMA (Preferred Reporting Items for Systematic Reviews and Meta-analyses) recommendations, we collected and evaluated experimental and epidemiological studies examining biological effects of exposure to static EF in humans (*n* = 8) and vertebrates (*n* = 40).

**Results:**

There is good evidence that humans and animals are able to perceive the presence of static EF at sufficiently high levels. Hair movements caused by electrostatic forces may play a major role in this perception. A large number of studies reported responses of animals (e.g., altered metabolic, immunologic or developmental parameters) to a broad range of static EF strengths as well, but these responses are likely secondary physiological responses to sensory stimulation. Furthermore, the quality of many of the studies reporting physiological responses is poor, which raises concerns about confounding*.*

**Conclusion:**

The weight of the evidence from the literature reviewed did not indicate that static EF have adverse biological effects in humans or animals. The evidence strongly supported the role of superficial sensory stimulation of hair and skin as the basis for perception of the field, as well as reported indirect behavioral and physiological responses. Physical considerations also preclude any direct effect of static EF on internal physiology, and reports that some physiological processes are affected in minor ways may be explained by other factors. While this literature does not support a level of concern about biological effects of exposure to static EF, the conditions that affect thresholds for human detection and possible annoyance at suprathreshold levels should be investigated.

**Electronic supplementary material:**

The online version of this article (doi:10.1186/s12940-017-0248-y) contains supplementary material, which is available to authorized users.

## Background

Static electric fields (EF) arise naturally in the environment, for example with the approach of storm clouds or through triboelectric charge separation on clothing, or they are artificially generated in association with technical processes or devices. The atmospheric static EF is generated between the positively loaded ionosphere and the negative ground. The EF strength, which measures about 0.1–0.3 kV/m at ground level, depends on the electric charges in the air, the season and the weather [1]. Higher levels of EF up to 500 kV/m are measured at the body from static charge on clothing [2]. Typical technical devices generating static EF are direct current (DC) transmission lines, cathode ray tube displays, trams and urban railways. High-voltage direct current (HVDC) transmission lines produce static EF up to 35 kV/m (±600 kV HVDC transmission line) [3], DC motors in railway systems generate up to 0.3 kV/m inside the train, and between 10–20 kV/m at a distance of 30 cm from cathode ray tube displays [4].

HVDC transmission line projects are becoming increasingly important to strategies to satisfy the worldwide growing demand for energy. With this technology, power grids are made more flexible and better able to sustain the shift from fossil fuels to renewable energy sources. Places where wind and solar energy or geothermal heat are collected are often far away from places where energy is needed. To connect generating sites to power grids and transmit energy over hundreds of kilometers, HVDC transmission lines offer a solution. When carried over long distances, DC is more efficient than alternating current (AC) because of lower power losses. A number of countries such as the United States, Canada, and Sweden already have multiple HVDC transmission lines, some of which have been operating for decades. At the same time, with the breakthroughs that have lowered the cost of the stations that convert AC to DC at one end of the line and DC to AC at the other end, more proposals for new lines have been made. As new projects are proposed, questions about the potential environmental effects of static electric and magnetic fields associated with the operation of these lines also have increased. While there are limit values for static magnetic fields recommended by the Council of the European Union [5] and the International Commission on Non-Ionizing Radiation Protection [6] (ICNIRP), the need for limits for exposure to static EF has not been suggested by health and scientific agencies. No static EF exposure limits have been recommended by ICNIRP [4] or the International Committee for Electromagnetic Safety (ICES) [7].

A non-systematic review performed for the National Radiation Protection Board (NRPB) in the United Kingdom by Kowalczuk et al. [8] evaluated 11 studies on the biological effects of static EF and concluded that the few experimental studies available did not provide evidence of adverse effects on human health. Furthermore, they concluded that the data available were not sufficient to establish a limit for human exposure to static EF. A 1997 review published by the U.S. Oak Ridge National Laboratory on the potential health effects of HVDC lines concluded that the data from 13 available studies on the effects of static EF were limited and that no mechanism has described how static EF could produce adverse biological responses [9]. The German Commission on Radiological Protection (SSK) and the International Agency for Research on Cancer (IARC) assessed the potential carcinogenicity of static EF. The SSK concluded that there is no evidence [10] and the IARC found inadequate evidence [11] for an association of static EF with cancer. The IARC also noted that no relevant data on the carcinogenicity of static EF in experimental animals were available [12] and static EF were classified in category 3 (not classifiable as to its carcinogenicity in humans). A review published in 2004 by the NRPB concluded that the most robust effect of static EF is cutaneous perception. However, only very few studies were considered in this review (*n* = 7) [13]. Two years later, the World Health Organization (WHO) evaluated seven studies regarding the effect of exposure to static EF on chronic or delayed health problems, but could not draw any conclusions based on this information [14]. Recently, the European Scientific Committee on Emerging and Newly Identified Health Risks (SCENIHR) stated that there is little information from representative population-based samples on thresholds for perception, annoyance, and other effects caused by static EF particularly with varying ion concentrations in the air [15].

It is generally agreed that in contrast to static magnetic fields, static EF do not enter the body [14]. Based on the physics of field interactions with the body, the static EF within the body from an external source is attenuated by a factor of approximately 10^−12^ [16]. According to the current knowledge, static EF can cause effects on the body via changes in the distribution of electric charges on the surface of the body. A sufficiently large surface charge density may be perceived through its interaction with body hair and by other effects such as spark discharges (micro-shocks). Micro-shocks can occur when a charged person who is well insulated from the ground touches a conductive grounded object, or when a grounded person touches a charged object that is well insulated from the ground [17]. The studies of these discharges are not within the scope of this review although such discharges in poorly designed exposure systems may have elicited responses from animals that were attributed by the investigators to other mechanisms of interaction.

The aim of this review was to evaluate whether the interaction of static EF with the body is limited to the surface or whether there is evidence that the fields may also act on biological functions and thus pose a potential risk to health. We collected, analyzed and evaluated published experimental and epidemiological studies on biological effects of static EF exposure in humans and vertebrates. Our review was prepared as a first important step to assess the quality and validity of published evidence, identify open research questions and, if indicated, to be used as a basis to recommend a limit value. It should be noted that charges produced by HVDC transmission lines on air molecules or aerosols affect the polarity and strength of the EF around the lines, but the potential effect of these charges *per se* are not considered in this review. For recent reviews of that research see Alexander et al. [18] and Perez et al. [19].

## Methods

### General information and literature search strategy

As prescribed by the PRISMA guidelines [20], we conducted a systematic literature search to identify relevant studies published from inception to July 2016 using our thematically specialized, open-access literature database EMF-Portal (www.emf-portal.org). The EMF-Portal is the most comprehensive scientific literature database on biological and health-related effects of electro-magnetic fields and was approved by the WHO as reference database (http://www.who.int/peh-emf/research/database/en/index1.html). It has been publicly available for more than 15 years and comprises currently 23,800 publications (November 2016). The EMF-Portal is used by scientists (e.g., [21–23]), government agencies (e.g., [24]), and academies of science (e.g., [25]). Relevant studies for inclusion in the EMF-Portal are identified on a daily basis with the aid of systematic search strategies in major literature databases such as Pubmed (years of coverage: 1946 to present), Cochrane Library (years of coverage: 1992 to present), and IEEE Xplore Digital Library (years of coverage: 1892 to present). Journals not listed in these databases are additionally screened and relevant publications are included in the EMF-Portal database. Furthermore, studies are identified through searches of the references listed in the studies found in the primary search. After identifying relevant publications, all studies entering the EMF-Portal are categorized according to basic characteristics such as exposure specifications (frequency, type of field) or type of publication (e.g., original research article, review, dosimetric study). For specific sources (e.g., HVDC) we add keyword categories of field exposures (static electric field, static magnetic field) to ensure best search results. Thus, every article is recorded with additional details based on a standardized scheme enabling us to perform highly specified searches. The only search term for our review was “static electric field” (for link to search string, see Additional file 1: Search strategy).

### Eligibility criteria and study selection

Eligibility criteria were determined using the *Participants/Population (P), Exposure (E), Control (C), Outcome measures (O) (PECO)* strategy [26]. Included in this review were experimental and epidemiological studies of humans or vertebrates *(*in vivo*)* (P) with exposures to static EF *(E)*. Valid controls were either a non-exposed group or a sham exposure condition *(C)* and considered outcome measures were biological effects *(O).* Further eligibility criteria were the indication of the static EF strength and an at least rudimentary description of the setup. Studies which referred to the description of the setup in another publication were also accepted. Articles were included if they were written in English or German. There was no restriction with regard to the year of publication. Studies which focused on air ions were only included if they exposed additional groups to static EF alone or explicitly stated that they examined the potential effects of HVDC lines. Review articles, editorials, commentaries and unpublished or clearly not peer-reviewed articles were excluded. Also excluded were studies on contact currents and micro-shocks as well as dosimetric studies, theoretical studies, and simulations.

Two authors (AP, SD) independently screened the studies for eligibility based on the inclusion/exclusion criteria. Articles were screened in two stages. First, titles and abstracts were reviewed. After the initial reviewing, full-text versions were obtained for possibly relevant articles. They were further examined to determine whether they clearly met the eligibility criteria. The two authors made a joint decision about the inclusion of the articles.

### Data extraction

The data from the experimental studies were extracted independently by two authors (AP, SD). The extraction protocol was defined and agreed upon before the start of the project. Extracted data included bibliographic data, the experimental model (human or animal), static EF strength, exposure duration, number of participants or animals, examined endpoints and outcomes. If the peer-review status of a study was unclear, a remark was made in Tables 1 and 2 (see column “remarks”). Disagreements and technical uncertainties were discussed and resolved between review authors (AP, SD, DS).

**Table 1:** Characteristics of individual human studies

Author, year	Exposed body part (number of subjects)	Exposure	Endpoints	Outcome	Remarks
Barron and Dreher [28]	whole-body (*n* = 10)	static EF of 1 kV/m alone (? or combined with air ions?) for approximately 1 h	physiological/health-related effects: blood parameters; respiration rate; pulse; blood pressure; brightness detection; reaction times; field awareness; muscular steadiness	no significant effect	also air ions tested; not clear if peer-reviewed, all subjects were pilots
Blondin et al. [29]	whole-body (*n* = 48)	static EF of <10 to 50 kV/m alone or in combination with air ions; 7–11 s per trial (several 100 trials during 1 day)	field perception	median perception threshold 45.1 kV/m without ions; high ion densities lowered the median threshold to 36.9 kV/m	
Chapman et al. [30]	arm (*n* = 16)	static EF of 30 to 65 kV/m; 7–11 s per trial (several 80 trials during 1 day)	field perception	subjects did not perceive static EF	also AC EF
Clairmont et al. [31]	whole-body	static EF of −40 to 40 kV/m alone or combined with different AC EF	field perception	perception threshold of static EF 10–20 kV/m; simultaneous AC EF lowered the threshold	main focus was technical
Haupt and Nolfi [35]	whole-body (*n* = 438)	static EF of approx. −16 to 21 kV/m and static MF of 22 μT of a HVDC line for at least 5 years	physiological/health-related effects: self-reported health issues (e.g., headache, depression, allergies, illness days)	no significant effect	cross-sectional epidemiological study
Odagiri-Shimizu and Shimizu [34]	human arm (*n* = 10–30 per condition)	static EF up to 450 kV/m with variable humidity	field perception	average perception threshold approx. 250 kV/m at 90% relative humidity and approx. 375 kV/m at 50% relative humidity; awareness decreased threshold and shaving of the arm made it impossible to detect the field up to 450 kV/m	
Oftedal et al. [32]	whole-body (*n* = 20)	static and AC EF and MF generated by visual display monitors, exposure for 2 weeks during work (at least 3 days/week, 2 h/day); average static EF of 2 kV/m without filter	physiological/health-related effects: self-reported skin symptoms	no significant effect	active and inactive filters nearly diminished static EF, but AC EF was only diminished by active filter; cross-over design
Skulberg et al. [33]	whole-body (*n* = 120)	static and AC EF generated by visual display monitors, without antistatic measure (VDU treatment with ionic liquid) static EF up to 95 kV/m, median value up to 0.23 kV/m; mean exposure duration 6 h/day	physiological/health-related effects: self-reported skin symptoms; general health symptoms; behavioral tests	skin symptoms could be reduced; no significant effect regarding general symptoms and behavior

**Table 2:** Characteristics of individual vertebrate studies

Author, year	Exposed animal (number of animals)	Exposure	Endpoints	Outcome	Remarks
Altmann [62] (article in German)	guinea pig (*n* = 5 per group, crossover design)	static EF of 0.42 kV/m and control only under Faraday conditions	organ parameters: oxygen consumption	metabolism was increased upon static EF exposure compared to a shielded environment in a Faraday cage	also studied AC EF; not clear if peer-reviewed
Altmann [49] (article in German)	white mouse, budgerigar and zebra finch, guinea pig (*n* = ?)	static EF of 1 kV/m (mouse, budgie, zebra finch (exposure duration regarding activity assessment was 1 h)), 0.42 kV/m (guinea pig); control only under Faraday conditions	perception/behavior: activity (mouse, budgie, zebra finch), organ parameters: free amino acids in liver or muscle tissue (guinea pig, mouse)	increased activity accompanied by higher oxygen consumption in guinea pig and higher level of free amino acids in all examined animals compared to a shielded environment in a Faraday cage	not clear if peer-reviewed
Angell et al. [44]	cattle (*n* = 200 in total; 2 herds of 50 animals under the HVDC line and 2 herds of 50 animals as controls)	static EF of 5.6 kV/m (mean value) and static MF (mean value of 4.1 nA/m^2^) and air ions (mean value of 13 k ions/cm^3^) from a 500 kV HVDC line continuously for 30 months	reproduction/development: productivity and general health status of cows and calves	no significant effect	experimental field study; same animals as in Ganskopp et al. [45]
Arzruny et al. [74]	rats (*n* = 20 per group, 2 groups)	static EF of 200 kV/m for 1 h	hematology/immunology: enzyme activity of type A phospholipases, lysophospholipase in erythrocyte and mitochondrial membranes	increase of enzyme activities in erythrocytes	
Atalay [59]	40 male guinea pigs (10 vertical, 10 horizontal, 20 control)	static EF of 1.9 kV/m (horizontal and vertical) for 9 h/day for 3 days	organ parameters: collagen synthesis (hydroxyproline levels in lungs)	increased hydroxyproline levels; vertical EF more effective than horizontal	
Atalay and Güler [63]	40 male guinea pigs (10 vertical, 10 horizontal, 20 control)	static EF of 0.58 kV/m (horizontal and vertical) for 9 h/day for 3 days	organ parameters: collagen synthesis (hydroxyproline level in liver)	decrease in hydroxyproline level; vertical EF more effective than horizontal	
Bailey and Charry [39]	male Sprague–Dawley rats (*n* = 5–20 per examined endpoint)	negative and positive static EF (3 kV/m, 12 kV/m) partially with air ions (5 × 10^3^/cm^3^, 5 × 10^5^/cm^3^) for 2, 18, or 66 h	perception/behavior: locomotor activity, rearing behavior, food and water consumption	no significant effect	main focus was on air ions
Bailey and Charry [38]	male Sprague–Dawley rats (*n* = 6–16 per experiment)	negative and positive static EF of 3 kV/m without or with air ions (5*10^5^/cm^3^) of the respective polarity; exposure duration 2, 18 or 66 h	brain/nervous system: serotonin level in the rat’s brain	no significant effect	main focus was on air ions
Charry and Bailey [40]	male Sprague–Dawley rats (*n* = 5–15 per experiment)	negative and positive static EF of 3 kV/m without or with air ions (2*10^5^/cm^3^) of the respective polarity; exposure duration 2, 18 or 66 h	brain/nervous system: norepinephrine and dopamine levels in the rat’s brain	no significant effect	main focus was on air ions
Cieslar et al. [70]	128 male Wistar albino rats (4 groups, 32 animals, respectively: 3 different EF strengths and a control group; 8 animals per time point: after 14, 28, 56 d of exposure or 28 d after a 56 d exposure)	static EF of 16, 25, or 35 kV/m for 8 h/day for 14, 28, or 56 days	organ parameters and hematology/immunology: oxidative stress (enzyme activity of catalase, superoxide dismutase, and level of gluthation peroxide, lipid peroxidation) in blood and liver	transient changes in prooxidant-antioxidant balance	
Creim et al. [47]	male Long Evans rats; 380 rats in total, in 9 different experiments (*n* = 40, except for one experiment with *n* = 20)	negative and positive static EF (−55 to 80 kV/m) with air ions (1.4*10^6^) or -/+ 55 kV/m with different air ion concentrations (2*10^3^ to 2.5*10^5^) for 1 h	perception/behavior: spatial avoidance behavior	rats significantly avoided the exposed area (≥55 kV/m) regardless of air ions	
Creim et al. [46]	male Long Evans rats; 56 rats in total, divided into 4 groups (*n* = 14 per group)	negative and positive static EF of 75 kV/m with air ions (2*10^5^) for 4 h/day for 5 days	perception/behavior: taste aversion learning	no significant effect	
Fam [50]	male and female ICR-SW mice (for exposure: 2 groups, male and female *n* = 21, respectively and for control *n* = 24/24 male/female; additionally progenies of these 4 groups were exposed and examined)	static EF of 340 kV/m for 18–22 h/day, parents approx. 5000 h (approx. 30 weeks) in total and their progenies 2000 h in total	perception/behavior: water consumption hematology/immunology: blood parameters reproduction/development: growth; fertility; number and weight of pups organ parameters: histomorphology of different organs	partially less water consumption; some changes in blood parameters (e.g., lower level of ß-globulins in males and higher in females, lower level of hemoglobin and reduced number of lymphocytes in females); weight of pups higher in first and fourth week after birth; no effect on number of pups; no effect on organ histomorphology	
Ganskopp et al. [45]	see Angell et al. 1990 [44]	static EF of −3.3–24.7 kV/m under the positive conductor and −35.6–6.9 kV/m under the negative conductor	perception/behavior: activity (nursing, drinking, walking, lying, standing); distribution in panel	more time was spent for nursing and drinking; fewer exposed than control cattle were observed in the central areas of pens but still most of the animals in the central area	experimental field study; same animals as in Angell et al. [44]
Gray et al. [37]	female B6C3F1 mice implanted with murine mammary adenocarcinoma (*n* = 44, 11 mice per group)	static EF of 450 kV/m; mice in restraining tubes while exposed for 4 h/day for 13 days; all mice received the chemotherapeutic agent adriamycin	therapeutic approach: tumor development	greater tumor regression	also static MF and switching EF
Güler and Atalay [64]	guinea pig (*n* = 40 in total; 3 groups: *n* = 10 vertical static EF, *n* = 10 horizontal static EF, *n* = 20 control group)	static EF of 1.9 kV/m for 9 h/day for 3 days (horizontal and vertical)	organ parameters: hydroxyproline levels of liver, lungs and kidney	increased hydroxyproline levels in liver, lungs and kidney; vertical EF more effective than horizontal	for liver, exactly the same values as in [65], possibly published twice
Güler et al. [65]	male guinea pig (*n* = 60, divided into 6 groups)	static EF of 0.9 to 1.9 kV/m for 9 h/day for 3 days (vertical and horizontal)	organ parameters: collagen synthesis (hydroxyproline level in the liver; histomorphological examination of liver slices)	0.9 kV/m vertical and horizontal EF decreased the amount of hydroxyproline in the liver, while the 1.9 kV/m vertical and horizontal EF increased the amount of hydroxyproline	for 1.9 kV/m exactly the same values as in [64] for liver, possibly published twice
Güler et al. [66]	male guinea pigs (*n* = 70 in total, 6 experimental groups and 1 control group with *n* = 10 each)	static EF of 0.3, 0.9, and 1.8 kV/m for 8 h/day for 3 days (vertical and horizontal)	organ parameters: lipid peroxidation, superoxide dismutase activity in spleen and testis	0.9 and 1.8 kV/m increased the lipid peroxidation and superoxide dismutase activity	
Güler et al. [67]	male guinea pigs (280 animals in total, 14 groups with *n* = 20 each)	static EF of 0.3, 0.6, 0.8, 0.9, 1, 1.35, 1.5, 1.8, and 1.9 kV/m for 8 h/day for 3 days (vertical)	organ parameters: oxidative stress in spleen and testes	oxidative stress increased with increasing static EF strength	also studied AC EF; main focus was on developing a theoretic neural network
Güler et al. [71]	male guinea pig (*n* = 140, divided into 14 groups, additional 20 animals for control group)	static EF of 0.3, 0.6, 0.8, 1, 1.35, 1.5, and 1.8 kV/m for 8 h/day for 3 days (vertical and horizontal)	organ parameters and hematology/immunology: oxidative stress: lipid peroxidation and enzyme activity of superoxide dismutase in guinea pigs’ plasma, liver, lungs and kidney	liver, kidney: significant increase of lipid peroxidation and SOD activity 1–1.8 kV/m; plasma: significant increase of lipid peroxidation and SOD activity 0.8–1.8 kV/m; lungs: significant increase of lipid peroxidation and SOD activity 1.35–1.8 kV/m; no difference between horizontal and vertical application of EF	also studied AC EF
Harutyunyan and Artsruni [75]	female albino rats (*n* = 72, divided into 3 groups)	static EF of 200 kV/m for 1 h or 6 h/day for 6 days	hematology/immunology: proteome changes in blood plasma	increased lysozyme activity after 1 h and 6 h/day for 6 days; decrease of globular proteins coinciding with clotting acceleration after 1 h; attenuation of clotting-dependent proteome modifications reflected with incomplete coagulation after 6 h/day for 6 days	
Harutyunyan and Sahakyan [76]	male albino rats (*n* = 10 per group, 2 experimental groups and 2 control groups)	static EF of 200 kV/m for 1 h or 6 h/day for 6 days	hematology/immunology: oxidative processes in red blood cells and cell number	after 1 h exposure: reduced number of red blood cells, increased oxidative stress and increased enzyme activities; after 6 days: increased number of red blood cells, oxidative stress parameters decreased or increased	
Kato et al. [48]	hindleg of anesthetized cats (*n* = 13)	static EF of 180–310 kV/m (both polarities) for 0.05, 0.2, 1.1, or 2 s for hair movement and for 1.5–3 min for action potential recording (sural nerve in 10 cats and sural, gastrocnemius, and articular nerves in 3 cats)	perception/behavior: hair movement; action potentials from afferent nerve fibers	cat hairs were attracted to the upper electrode when exposed to static EF of 180 kV/m or more; afferent impulses were evoked from hair receptors that were probably stimulated by the hair movement; deeper receptors were not affected by the EF	
Kellogg and Yost [41]	female NAMRU mice (*n* = 200 in total, 8 groups, *n* = 25 each, in results partially less animals stated)	static EF of 1.8 kV/m plus 2*10^3^ air ions/cm^3^; static EF of 2.4 kV/m plus 2*10^5^air ions/cm^3^; static EF of 2 kV/m (both polarities, respectively); 2 control groups; exposure started with approx. 6 weeks until end of life (except for 6 h every 2 weeks when cages were cleaned)	hematology/immunology: mortality	no effect found between static EF alone versus control group	main focus was on air ions; extension of [42, 43]; but unclear why different field strengths are reported. Duplicative publication of results from Kellogg et al (1985a, b) [42, 43]. In second year proteus vulgaris infection in all exposure groups caused severe gastroenteritis, splenic hypertrophy, occasional purulent salpingitis, and death.
Kellogg et al. [42]	female NAMRU mice (*n* = 200 in total, 8 groups, *n* = 25 each, in results partially less animals stated)	static EF of 1.75 kV/m plus 2*10^3^ air ions/cm^3^; static EF of 1.98 kV/m plus 2*10^5^ air ions/cm^3^; static EF of 2 kV/m (both polarities, respectively); 2 control groups; exposure started with approx. 6 weeks until end of life (in this article only first year reported)	hematology/immunology: blood parameters (serotonin, glucose, cholesterol, urea nitrogen, globulin) every three months	increased mean glucose level in positive EF and decreased urea nitrogen in negative EF compared to overall mean values	main focus was on air ions; results for first year of a two year study, see [43] for second year; possibly mild vitamin deficiency
Kellogg et al. [43]	see Kellogg et al. [42]	see Kellogg et al. [42] (in this article only second year reported)	hematology/immunology: blood parameters (serotonin, glucose, cholesterol, urea nitrogen, globulin); survival rate organ parameters: organ parameters (weight, histologic changes)	animals in static EF lived longer (no significance given) and increased mean glucose level in positive EF compared to overall mean value; no effect on organ weights	main focus was on air ions; results for second year of a two year study, see [42] for first year; possibly mild vitamin deficiency. In second year, proteus vulgaris infection in all exposure groups caused severe gastroenteritis, splenic hypertrophy, occasional purulent salpingitis, and death.
Krueger et al. [73]	NAMRU mice (*n* = 90–220 mice per group, 12 groups)	static EF of ca. 0.1 kV/m (ion depleted air or with 2.7-5*10^3^ air ions) or static EF of 4–6 kV/m (ion depleted air or with 2.3-5*10^5^ air ions) for up to 11 days	hematology/immunology: mortality after influenza infection	no significant effect	main focus was on air ions
Lott and McCain [58]	male Sprague–Dawley rats (in total, *n* = 60)	surface electrodes measured EEG (*n* = 30) electrodes implanted in the brain measured hypothalamic activity (*n* = 30); 15 rats of each group were exposed to a pulsed EF and 15 to a static EF of 10 kV/m for 50 min during EEG recording; experiments were performed under anesthesia	brain/nervous system: brain activity and hypothalamic activity	increased brain activity measured with surface electrodes and decreased hypothalamic activity (implanted electrodes); when EF was turned off, brain activity returned back to baseline before exposure	
Marino et al. [78]	female Swiss Ha/ICR mice (*n* = 5 per EF strength and exposure duration)	2.7 kV/m and 10.7 kV/m (parallel to earth’s surface); 5.7 kV/m (perpendicular to the earth’s surface) for 7, 14, or 21 days	hematology/immunology: concentration of serum proteins (albumin, alpha-, beta-, gamma-protein)	beta globulin increased under parallel and decreased under perpendicular exposure; albumin vice versa; gamma globulin increased under perpendicular exposure	
Marino et al. [69]	male Sprague–Dawley rats (7 different exposure groups with *n* = 12–20, corresponding control groups with *n* = 8–23); female Swiss Ha/ICR mice bearing Ehrlich ascites tumor cells (*n* = 7 exposed, *n* = 6 control)	rats: static vertical EF of 0.6, 2.8, 5.6, 19.7 kV/m and horizontal static EF of 0.3, 2.8, and 9.8 kV/m for 30 days; mice: horizontal static EF of 8–16 kV/m for 14 days	hematology/immunology (rats): body weight, serum protein concentration organ parameters (rats): histomorphology of different organs (lungs, liver, kidney), general examination genotoxicity (mice): chromosomal aberrations in tumor cells	rats: secondary glaucoma (only vertical EF); altered serum protein concentration; no effects on organ histomorphology; mice: production of chromosomal abnormalities in tumor cells	
Mayyasi and Terry [51]	King-Holtzman rats (in total, *n* = 240, different age and sex groups; additional groups to test the influence of noise)	static EF of 1.6 and 16 kV/m for 5 h	perception/behavior: behavior, learning	improved learning (less errors) and better swimming performance	
Mitchell et al. [79]	female mice (Swiss Ha/ICR) (*n* = 8–12 per group, data taken from results, number not stated in methods)	static EF in the range of 8 to 16 kV/m up to 15 weeks (horizontal and vertical)	genotoxicity: chromosomal aberrations in tumor cells	increase in the percentage of abnormal chromosomes after 2 weeks of horizontal exposure; disappearance of the aberrant chromosomes after extended exposure	extension study of Marino et al. [69]
Möse and Fischer [52] (article in German)	white mice (*n* = 5 for locomotor activity and food/water consumption), *n* = 10 for rectal temperature, *n* = 12 for litter frequency, no number given for oxygen consumption of liver)	static EF of 23.8 kV/m for 15 days up to approx. 4 months (litter frequency) (mostly 15 to 20 days)	perception/behavior: locomotor activity, food/water consumption, rectal temperature reproduction/development: litter frequency organ parameters: oxygen consumption of liver pieces	significant increase of locomotor activity, food and water consumption and rectal temperature; initially normal number of offspring, over the later period complete absence of offspring; no effect on oxygen consumption of liver pieces	not clear if peer-reviewed
Möse et al. [56] (article in German)	rat and guinea pig (*n* = ?)	static EF of 23.8 kV/m for 6 days	organ parameters and brain/nervous System: serotonin concentration in uterus of rats, brain and ileum of guinea pigs	reduced serotonin levels in brain and uterus, increased serotonin levels in ileum	not clear if peer-reviewed; contradictory statements if brain was taken from rats or guinea pigs
Möse et al. [68] (article in German)	rat (uterus) and guinea pig (intestine) (*n* = ?)	static EF of 23.8 kV/m for 3–21 days	organ parameters: ability of isolated smooth muscles (intestine and uterus) to react to stimulating drugs	smooth muscles reacted less to stimulating drugs	not clear if peer-reviewed
Möse et al. [60] (article in German)	male NMRI mice (*n* = 25 per group, 3 groups: static EF, control group under ambient conditions, control group in Faraday cage)	static EF of 23.8 kV/m for 8 days	organ parameters: oxygen consumption of isolated liver pieces	increased oxygen consumption under static EF exposure and decreased consumption under Faraday conditions in comparison to control group	not clear if peer-reviewed
Möse et al. [72] (article in German)	NMRI-Hahn mice (*n* = 10–15 per EF strength and condition)	static EF of 0.04, 0.2, 1, 5 and 24 kV/m for 15 days; in addition to control group under laboratory conditions also control in Faraday cage; mice were immunized with sheep erythrocytes at day 7 and 11 of exposure	hematology/immunology: immunization level (plaque formation on spleens, spleen weight, number of spleen cells, haemagglutination titers)	immunization level increased under static EF exposure compared to a shielded environment in a Faraday cage	not clear if peer-reviewed
Okudan et al. [61]	male and female Wistar rats (progenies of 3 dams, already exposed during pregnancy); (*n* = 10 for static EF, *n* = 7 for control)	static EF of 10 kV/m for 28 days (14 days *in utero* and 14 days postnatal)	organ parameters: bone mineral density; bone mineral content	decrease in bone mineral content and bone mineral density	also studied AC EF
Sahakyan et al. [77]	female rats (*n* = 120; divided into 2 groups)	static EF of 200 kV/m for 6 h/day for 6 days	hematology/immunology: lipid-protein interactions in rat erythrocyte membranes	redistribution of membrane surface charge, conformational alterations of membrane integral proteins, increase of the immersion degree of the peripheral proteins	incomplete reference list
Xu et al. [57]	male ICR mice (*n* = 90, divided into 2 experimental groups and 1 control group of 30 animals each)	static EF of 2.3–15.4 kV/m (experimental group 1) and 9.2–21.85 kV/m (experimental group 2) from a HVDC line for 35 days except for raining and low temperature days	brain/nervous System: cognition: learning, memory; glutamate and GABA concentrations in the brain	impaired memory abilities	

The single epidemiological study was extracted by a third author (DD). The extracted data included population characters, such as study size, age of participants, rate of participation, exposure source, examined endpoints and outcome.

### Study appraisal

To assess the internal validity (i.e., the extent to which a study is free from risk of bias in design, conduct and analysis) and the overall quality of the included studies, we used a modified approach recommended by the National Toxicology Program’s Office of Health Assessment and Translation (OHAT) [26, 27]. The OHAT risk of bias rating tool consists of a set of questions and provides detailed instructions how to evaluate methodological rigor in human and animal studies with a focus on environmental health and toxicology. Depending on study design (experimental human, cross-sectional or experimental animal), up to 10 methodological criteria were applied to rate studies for biases in selection, performance, detection, attrition/exclusion, or selective reporting. Besides the items recommended in the OHAT handbook published in 2015 [26], we included an additional criterion to rate whether the study design of experimental human and experimental animal studies accounts for confounding or modifying variables. This criterion is essential for the evaluation of studies with exposures to EMF because missing control for confounders like the presence of air ions, ozone, corona, or noise considerably lower the certainty that the reported exposure effects are due to static EF. Two authors (AP, KS) independently evaluated methodological criteria of all studies meeting selection criteria according to the following ratings: “++” definitely low risk of bias; “+” probably low risk of bias; “-” probably high risk of bias, or “--” definitely high risk of bias. Disagreements between the two authors were discussed and resolved by consensus. To reach conclusions about the overall risk of bias of individual studies we used the OHAT approach for determining tiers of study quality [26]. OHAT outlines a 3-tier system with “key” risk of bias elements being defined on a project-specific basis. The three critical risk of bias elements which were given the highest weight in determining study quality in this evaluation were (1) study design that addressed confounding/modifying variables (2) confidence in the exposure characterization, and (3) confidence in the outcome assessment. Placement of a study into one of three study quality categories (1^st^ tier, 2^nd^ tier, or 3^rd^ tier) was contingent on the rating of these three key risk of bias criteria and proportions in the rating of the remaining criteria (for more detailed descriptions and example classifications see Additional file 1: Table S1). A meta-analysis of numerical results was not possible for this review due to the substantial heterogeneity between studies in terms of differences in study populations, study protocols, study types and endpoints. In addition, attention was given to effects for which replication by independent investigators had been reported.

## Results

### Study selection

**Fig. 1:**
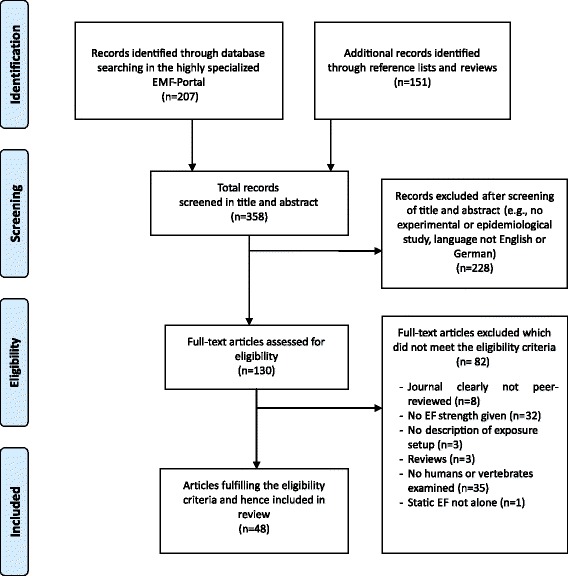
Flow diagram of literature search, eligibility and inclusion process. Adapted from Moher et al. [20]

The systematic search identified 358 articles that matched the search criteria. After screening the title and abstract, 228 articles were excluded for various reasons (e.g., secondary literature, dosimetric articles or not dealing with static EF on biological systems). The full text was obtained for the remaining 130 articles to check for eligibility to be included in our analysis. Of these, 82 articles were excluded for the following reasons: not dealing with humans or vertebrates (*n* = 35), static EF strength not indicated (*n* = 32), journal clearly not peer-reviewed (*n* = 8), no description of exposure setup (*n* = 3), reviews (*n* = 3), or exposure not with static EF alone (*n* = 1). Forty-eight articles fulfilled the aforementioned eligibility criteria and were included in this review (see also Fig. 1). Of these, one article reported an epidemiological study in humans (cross-sectional); all other articles reported experimental studies (seven experimental human trials, 40 experimental animal studies).

**Fig. 2:**
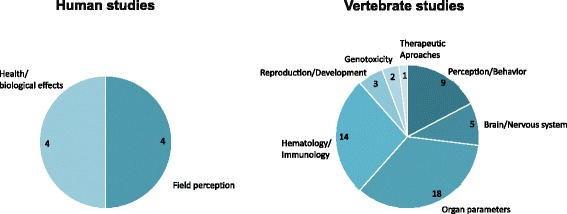
Endpoints in human and vertebrate studies. The numeric values indicate the number of studies which examined the listed endpoints. Note that some animal studies examined two or more endpoints and are therefore listed more than once in the pie chart

The endpoints evaluated in human studies were field perception and physiological/health-related effects upon exposure to static EF, while the majority of animal studies investigated histological/biochemical organ parameters and hematologic/immunologic functions. Perception/behavioral responses were the third most studied endpoint in animal studies. Other endpoints examined in animal studies related to brain/nervous system, reproduction/development, genotoxicity, and therapeutic approaches (Fig. 2).

Human studies on field perception are discussed in greater detail because international scientific committees have stated that field perception is the most robust effect and recommended the collection of further data [11, 15].

### Study appraisal

**Fig. 3:**
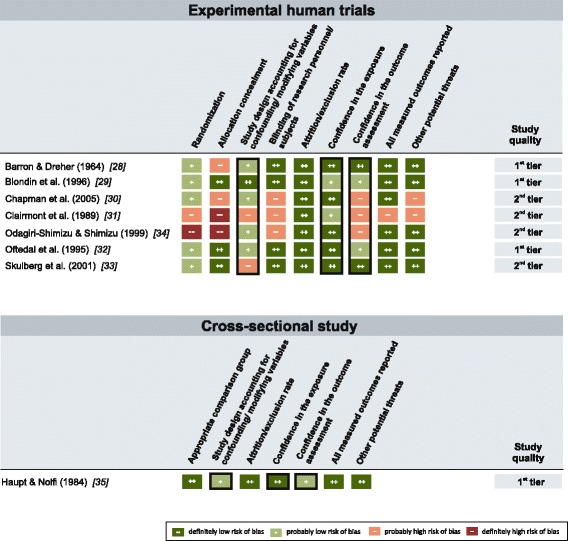
Quality assessment for human studies. Risk of bias ratings for seven human experimental trials and one cross-sectional study. Criteria ratings served as basis for the assignment of individual studies to one out of three study quality categories (1^st^ tier, 2^nd^ tier, 3^rd^ tier). Black frames indicate key risk of bias criteria

**Fig. 4:**
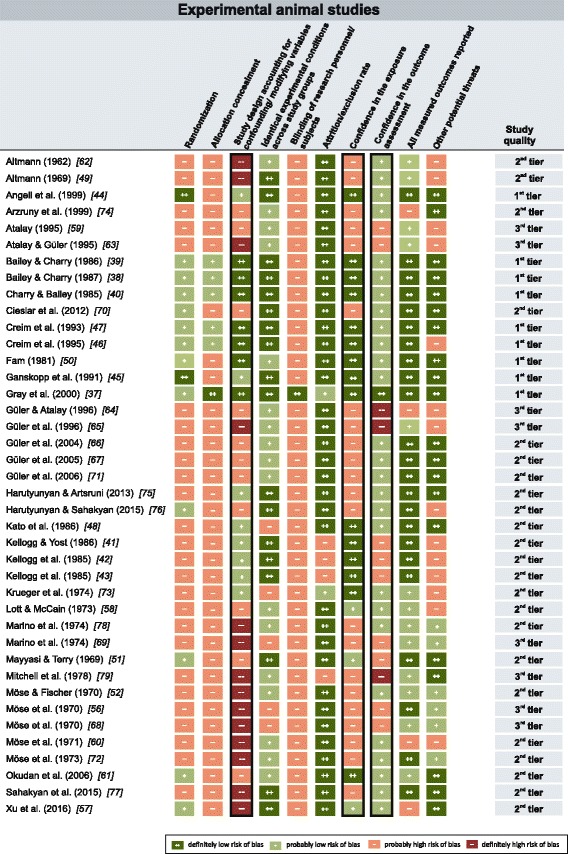
Quality assessment for animal studies. Risk of bias ratings for 40 experimental studies in vertebrates. Criteria ratings served as basis for the assignment of individual studies to one out of three study quality categories (1^st^ tier, 2^nd^ tier, 3^rd^ tier). Black frames indicate key risk of bias criteria

The OHAT risk of bias rating tool was used to evaluate risk of bias in design, conduct and analysis in individual human and animal studies and to reach conclusions about their overall quality (Figs. 3 and 4).

Overall, human studies were generally less susceptible to risk of bias than were animal studies (Fig. 3). Four out of eight (50%) human studies were placed in the “1^st^ tier”; the remaining four studies (50%) were placed in the “2^nd^ tier”. Many of the animal studies suffered from severe methodological flaws (Fig. 4). Out of the 40 animal studies, only nine (22.5%) were placed in the “1^st^ tier”, 23 (57.5%) were placed in the “2^nd^ tier” and eight (20%) studies were placed in the “3^rd^ tier”.

A randomized method for the assignment of subjects or animals to study groups was not reported in two human studies and in more than half of the animal studies (*n* = 26). Also, inadequate allocation concealment introduced a substantial risk of bias in a large number of studies (four human studies and 34 animal studies). A major potential threat to internal validity was missing or insufficient control for possible confounders (e.g., ozone, air ions, noise, or micro shocks) in two human and 25 animal studies. Blinding of the research personnel and participants during exposure was not adequately addressed in four human studies, and only one animal study was explicitly conducted under blinded conditions. In more than half of the animal studies (*n* = 22), the static EF strength was not explicitly verified through measurements or simulations (missing confidence in the exposure characterization) and can therefore be considered a risk of bias in these studies. Confidence in the outcome assessment was limited through the use of insensitive instruments or non-validated methods in three human and 12 animal studies.

### Static EF influences on humans

Seven experimental studies [28–34] and one epidemiological study [35] examined the effects of static EF in humans (field perception and physiological/health-related effects) (Table 1). All but the epidemiological study focused on acute, short-term effects of static EF. The size of the study populations was between 10 and 58 participants in the experimental studies; 438 participants were involved in the epidemiological study. Exposure levels ranged between −40 kV/m and +450 kV/m (+ and – indicate polarity of the EF).

#### Field perception

Field perception experiments provided evidence that detection thresholds for static EF are much lower for whole-body exposure [29, 31] than limb exposure (e.g., arm and forehand) [30, 34]. Because these effects were confirmed by independent investigators, they can be considered as replicated. Blondin et al. [29] found that under whole-body exposure (static EF strength up to 50 kV/m, 7–11 s/trial) the median detection threshold of seated and grounded male and female subjects was 45.1 kV/m for a static EF. Approximately, 5% of the participants could detect a static EF below 20 kV/m, 33% of the subjects detected a static EF below 40 kV/m and 66% detected fields below 50 kV/m. Co-exposure to air ions with ion current densities of 60 nA/m^2^ did not affect detection thresholds. When air ions in high concentrations (120 nA/m^2^) were added, the sensitivity was increased, permitting subjects to detect the EF at lower field strengths. Here, the median value was 36.9 kV/m, with some participants being able to perceive weaker fields of 10 kV/m or less. The authors estimated that the detection thresholds reported for seated subjects would be lowered if they were standing. Clairmont et al. [31] made observations under a hybrid power line (AC/DC) and found that when static EF (up to 40 kV/m) were combined with AC EF, detection thresholds were lower than what would be expected for static or AC EF alone, i.e., the combination of both greatly enhanced the perceived sensation. For static EF alone, an average detection threshold of 20 kV/m was estimated from the given data. Furthermore, 20% of the participants rated a static EF alone of 15 kV/m as “just perceptible”. However, this study had some methodological flaws (e.g., no appropriate control for confounders, subjects not grounded and not naive as to the purpose of the study, see Fig. 3).

Two further experiments were conducted under partial-body exposure where only the participants’ arm was exposed to static EF [30, 34]. Odagiri-Shimizu and Shimizu [34] used EF strengths of up to 450 kV/m and showed that the subjects were able to perceive static EF above 250 kV/m on their forearm when the relative humidity was 90%. When the humidity was set to 50%, the detection threshold increased to about 375 kV/m. Furthermore, when the volunteers knew that the field was on (awareness), detection of the static EF was facilitated. When the arm was shaved, the participants were no longer able to perceive a static EF at intensities up to 450 kV/m. This suggests that the perceived sensation is dependent on body hair. A similar study was conducted by Chapman et al. [30]. They exposed only the forearm of the subjects to a static EF (between 30 and 65 kV/m, 7–11 s/trial), but none of the subjects was able to perceive the fields. However, the maximum applied EF strength was much lower (65 kV/m) than in the study by Odagiri-Shimizu and Shimizu [34]. The authors concluded that the applied field strengths were too low to be detected under partial-body exposure and that the exposed body surface area could play a crucial role in the detection of static EF.

The striking differences in detection thresholds under whole-body and partial-body exposure are most parsimoniously explained by the higher EF on some parts of the body with whole-body exposure. The presence of a person in an EF will perturb the uniformity of the field. Field lines then concentrate on body parts closest to the EF source, i.e., the field increases at the top of the body (e.g., head/shoulder) about a factor of 13–18 while it decreases at lower body parts (e.g., arms and legs) relative to upper body parts [36]. Such a field increase in the head/shoulder region should facilitate the perception of the field. The notable field increases may also explain why both Blondin et al. [29] and Clairmont et al. [31] reported that some people are able to detect static EF at field strengths of 10 kV/m and even below that level. Field perturbation occurs much less when only the forearm is exposed because of the comparatively flat shape of the arm. This could explain the much higher EF strengths required for detection performance under partial-body exposure in the studies by Odagiri-Shimizu and Shimizu [34] and Chapman et al. [30]. Other factors that may influence perception of static EF are the density and length of hair on the body. In addition, in human studies, a lowering of detection thresholds in experimental situations might occur where awareness as to possible exposure and lack of distracting/confounding stimuli prevail.

#### Physiological/health-related effects

In addition to field perception experiments, we identified four other studies which examined physiological and health-related effects in humans upon exposure to static EF. The results of these studies have not been replicated, yet.

One of the experimental studies on skin symptoms among visual display unit users found that facial skin complaints might be caused by a combination of exposure to static EF (0.23 kV/m on average for 6 h/day) and high dust concentrations [33], while a study by Oftedal et al. [32] could not find a relation between skin symptoms and exposure to static EF (2 kV/m on average for 2 h/day). Furthermore, it could not be shown that static EF (1 kV/m) alter psychomotor and physiological functions in a group of pilots [28]. In the only epidemiological study in this field, Haupt and Nolfi [35] considered potential health effects in relation to residential proximity to a HVDC transmission line. Examined endpoints were symptoms of discomfort (e.g., headache, depression, eye irritation), health status, number of physician visits, and illness days. People who had lived in close proximity (less than 225 m) to the 400 kV Pacific Intertie HVDC transmission line in California for at least 5 years were included in this study (*n* = 438). Static EF strengths were approximately 21 kV/m under the positive pole and −16 kV/m under the negative pole at ground level according to measurements on a similar test line. Static magnetic fields and air ions were also present. The results showed no statistically significant association between exposure to the HVDC transmission line and perceived health problems among adjacent residents.

### Static EF influences on vertebrates

There were 40 studies of vertebrates eligible for this review; mainly rats and mice were examined in these studies. One study had a therapeutic purpose [37]. This was the only study that was explicitly conducted under blinded conditions. Seven studies focused on the effects of air ions [38–43, 73]; these studies were included in this review because they also tested a static EF alone. An additional four studies investigated exposures to EF from a HVDC line [44, 45] or a simulated HVDC environment [46, 47]. In these studies, the animals were co-exposed to air ions and static EF. It is, however, not possible for the latter studies to clearly distinguish between the possible independent contributions of air ions and static EF to the examined endpoints.

The discussion of the animal studies is organized below according to the examined endpoints. Some of the studies examined more than one endpoint and are therefore discussed in several sections.

A considerable number of studies indicated static EF influences on e.g., behavior, metabolism or blood parameters. Some authors hypothesized from their results that static EF may directly interact with biologic systems and alter cell functioning, but evidence for a direct effect on tissue was not provided in the literature. The results of these studies should thus be considered from the point of view that none of these studies was designed to determine to what extent these responses might reflect a direct field interaction with interior tissues or an indirect, internal response due to sensory stimulation of the body surface.

#### Perception mechanism/Behavioral responses

Similar to what has been shown in humans, Kato et al. [48] found evidence that body hair is involved in the perception of static EF by cats. The authors recorded afferent impulse discharges of hair receptors when the anesthetized cats were exposed to static EF (180–310 kV/m). The stronger the EF, the wider was the angle of the hair movement. In addition, more action potentials were triggered with increasing EF strength. Deeper skin receptors were not affected. This effect, therefore, is consistent with electrostatic forces causing hair movement that leads to sensory stimulation and detection of static EF.

A further eight studies investigated behavioral responses of vertebrates to static EF exposure [39, 45–47, 49–52], but the results have not been clearly replicated by separate laboratories. Locomotor activity, avoidance behavior and food and water intake were mainly examined in mammals. Birds were studied besides mice in one study [49]. The studies differed greatly regarding the applied EF strengths (1–340 kV/m), exposure duration (1 h to several months) and the numbers of treated animals (10 to 360). Additionally, the provided documentation often did not allow us to appropriately assess the quality of the experimental setup and methods e.g., [49, 51, 52]. Despite these methodological limitations and the limited data available, there is good evidence that static EF can be detected and elicit behavioral responses in vertebrates probably due to sensory stimulation of the skin and body hair. In rodents and some other animals, the vibrissae are important mechanosensory receptors that are sensitive to tactile stimulation, which modulate a wide variety of behaviors, and this helps explain why secondary physiological responses to tactile stimuli, including static EF should be expected [53]. Besides hair movement as a physical mechanism for the detection of static EF, it can be further hypothesized that high EF strengths may lead to an ionization of air ions and ozone production, known as the corona effect. The well-developed sense of smell in animals also may help them perceive the simultaneous presence of ozone and initiate a response to the static EF.

Three of these studies reported that static EF (between 1 kV/m and 23.8 kV/m, between 1 h and 20 days) have a stimulating effect on the locomotor activity [49, 51, 52]. Studies by Altmann and Möse on locomotor activity [49, 52] were motivated by previously reported results of positive and stimulating effects of both static EF and air ions on humans and animals (e.g., improvement in cognitive performance in humans, general health promotion of human and cows [54, 55]). These findings were not confirmed by Bailey and Charry [39]. As part of a study of air ions in which groups of animals were exposed to static EF alone (3 kV/m or 12 kV/m for 2, 18 or 66 h), the authors found no influence of static EF on two continuous measures of motor activity in rats.

Two studies investigated avoidance behavior in rats [46, 47]. Creim et al. [47] showed that rats avoided static EF (between 55 and 80 kV/m for 1 h), regardless of the presence of air ions. This behavior was found to be dose dependent with higher field strengths inducing greater field avoidance. In a later study, Creim et al. [46] failed to induce taste-aversion learning in rats in exposed environments (75 kV/m, 4 h/day for 5 days). The authors speculated that avoidance behavior observed in the earlier study was likely prompted by a response to external sensory stimulation, i.e., the perception of the static EF on the fur. The second study, however, indicated that internal stimulation such as gastrointestinal distress did not occur as a consequence of exposure.

Exposure effects on food and water intake were investigated in three studies [39, 50, 52]. Bailey and Charry [39] (with exposures at 3 kV/m or 12 kV/m for 2, 18 or 66 h) did not report any effect in rats, but the other two studies by Fam [50] (exposure at 340 kV/m for 18–22 h/day for 30 weeks) and Möse and Fischer [52] (exposure at 23.8 kV/m for 15 to 20 days) found altered food and water intake in mice.

Various aspects of cattle behavior were investigated in an experimental field study by Ganskopp et al. [45]. They tracked the animals’ activity and distribution under exposure to the static EF of a 500 kV HVDC transmission line and concluded from the data that they do not provide evidence that a static EF or other aspects of the HVDC electrical environment altered the behavior of cattle.

#### Effects on the brain and nervous system

Five studies were identified that investigated the effects of static EF on the nervous system of rats and mice [38, 40, 56–58], but the results of these studies have not been replicated thus far. Exposure durations were between 50 min and 35 days and the applied EF strengths varied between 3 kV/m and 23.8 kV/m. Study populations had a size of 5 to 30 animals per group.

Four of these studies examined various neurotransmitter concentrations in the brains of rodents, but the results were inconsistent [38, 40, 56, 57]. Möse et al. [56] reported significantly reduced serotonin levels in the brain of guinea pigs that had been exposed to a static EF (23.8 kV/m for 6 days). They hypothesized an association between metabolic changes – possibly triggered by an activating action of static EF and air ions – and the decrease in serotonin level (see section Histological and biochemical organ parameters). In contrast, three other studies found no changes in neurotransmitter concentrations. Bailey and Charry [38] and Charry and Bailey [40] reported that norepinephrine, dopamine and serotonin concentrations were not affected in rats’ brains after the animals were exposed to a static EF (3 kV/m for 2,18 or 66 h). Xu et al. [57] tested spatial learning and memory abilities of mice previously exposed to a static EF (between 2.3 and 21.85 kV/m for 35 days) beneath a HVDC line in the ambient environment. They did not find changes in glutamate and GABA levels which have been associated with learning and memory abilities in some other studies. However, the authors found that mice which were exposed at the highest field strengths showed behavior suggestive of impaired memory ability in a water-filled maze. Because changes in neurotransmitter concentrations did not account for the differences in performance between exposed and control mice, Xu and his co-workers hypothesized that static EF might suppress the expression of receptors which are involved in memory formation.

Lott and McCain [58] found changes in electro-encephalographic (EEG) recordings of rats under the influence of static EF (10 kV/m for 50 min). They showed that the EEG was modified (increase in cortical brain activity and reduced hypothalamic activity) when switching the static EF on and returned back to baseline values when the field was turned off again. The authors suggested that the increased general brain activity under exposure conditions lowered the activity of the hypothalamus. They interpreted their data showing a neuronal correlate for the rats’ ability to detect static EF, with the hypothalamus being a putative electro-sensitive region. Potential confounding due to coupling of the external field to the electrode, especially when the field was turned on or off during recording the electrical activity of the brain, or that the EEG recording reflected sensory stimulation of the skin or fur was not considered or discussed.

#### Histological and biochemical organ parameters

In total, 18 studies examined various histological and biochemical parameters (metabolic activity, histological effects, collagen synthesis, oxidative stress and bone density) in different organs in rodents. No studies by independent investigators attempted to replicate the reported results. Organ parameters were the main focus in ten studies [59–68], whereas in other more comprehensive studies, organ parameters were only one of the endpoints evaluated among others (e.g., [43, 49, 50, 52, 56, 69–71]). The number of animals per group differed between 5 and 32. The applied field strengths ranged from 0.42 to 340 kV/m and exposure durations varied between 3 days and 2 years. A good number of studies reported effects on several histological and biochemical parameters upon exposure to static EF, but most of these studies had several methodological flaws (see Fig. 4). Some of the evaluated studies also lacked clear hypotheses as to the choice of examined endpoints or a discussion on the relevance of their results for possible health effects. The reported effects on metabolic functions and collagen synthesis were mainly discussed in terms of direct cell-field interactions. Some studies emphasized the beneficial effects of static EF on metabolism compared to animals held in an environment shielded by a Faraday cage.

Five studies – all conducted by Altmann and Möse – reported that static EF have a stimulating effect on metabolic activity in rodents [49, 56, 60, 62, 68]. Static EF strengths in the studies by Altmann were 0.42 kV/m [62] and 1 kV/m [49], respectively, while in the studies by Möse and colleagues the animals were exposed at a field strength of 23.8 kV/m [56, 60, 68]. Only one study with exposures at 23.8 kV/m did not find such a stimulating effect on metabolism [52]. It was speculated that altered metabolic functions may be the result of direct effects of static EF and air ions. Altmann [49] and Möse et al. [60] suggested a mechanism through which static EF act on cell functions by modifying bioelectrical potentials which in turn lead to increased cellular respiration. Möse and colleagues discussed that absorbed air ions may induce a serotonin release in the brain [56] or a shift in the metabolic activity of organs [68]. However, the authors did not consider the possibility that the responses reported also could have been indirect effects resulting from external sensory stimulation by the static field.

Additionally, one of these studies reported that mice which were kept in a Faraday cage (which blocks both ambient static and low frequency EF), had a lower oxygen consumption compared to the control group under ambient conditions [60]. According to the authors, lowered oxygen consumption, i.e., decreased metabolic activity, of rodents held in a Faraday cage indicates that these animals were disadvantaged by the absence of both static EF and air ions (see also section Hematology and immunology, Möse et al. [72]). The authors speculated that shielding from the natural EF, as occurs in most buildings, may have adverse effects on health.

A direct interaction between static EF and tissue proteins was proposed in several studies, all conducted by the same research group, which examined collagen synthesis in guinea pigs based on measurements of hydroxyproline levels in various organs [59, 63–65]. Güler, Atalay and colleagues chose to examine collagen, being the most abundant protein in vertebrates. Low EF strengths (between 0.58 and 0.9 kV/m with exposures of 9 h/day for 3 days) [63, 65] led to a reduction in the tissue hydroxyproline concentration, while exposure at 1.9 kV/m for the same exposure durations [59, 64, 65] led to an increase in hydroxyproline levels. The authors suggested that static EF influences on protein biosynthesis may be the result of penetration of static EF into the tissue. However, there was no attempt by the authors to explain why decreases and increases of hydroxyproline levels vary unpredictably as a function of EF strength. It was merely suggested that there could be a threshold below and above which decreases and increases of hydroxyproline concentration are triggered, respectively. In all four trials, the vertical field resulted in a stronger effect than the horizontal EF and this finding was confirmed in an additional histological examination of the liver with decrease and increase in collagen fibers being only observed under vertical static EF exposure [65].

Four more recent studies on rodents by some of the same investigators who proposed static EF effects on proteins also reported that exposure to static EF can induce oxidative stress in various organs [66, 67, 70, 71]. The authors of these studies did not discuss the potential mechanisms of action by which oxidative stress could be induced and it remains unclear how or why static EF could cause this response.

The study by Okudan et al. [61] provided some evidence for the influence of static EF (10 kV/m for 28 days) on bone density and mineral content after exposure of fetal and newborn rats, although the basis for this finding is unclear.

Finally, studies in three separate laboratories investigated the possible effects of static EF (with exposures between 0.6 and 340 kV/m for at least 30 days up to 30 weeks) on the histological appearance of diverse organ systems of rats and mice [43, 50, 69]. None of these studies found histological abnormalities in organs such as lungs, liver, kidney or testis. However, Marino et al. [69] reported that some of the animals developed secondary glaucoma (an eye disease). This unexpected effect was only observed in rats exposed to vertical static EF, but not in those exposed to horizontal fields or in the control group. The authors considered it likely that glaucoma was induced by static EF. However, no other study in the evaluated literature examined or reported any effect of static EF on eyes.

#### Hematology and immunology

Fourteen studies evaluated hematologic and/or immunologic parameters. Again, the results of these studies have not been replicated by independent investigators. Four studies focused on the effect of air ions [41–43, 73], the remaining ten studies examined whether the static EF itself affected these parameters [50, 69–72, 74–78]. The applied static EF field strengths varied between 0.04 kV/m and 340 kV/m and animals were exposed between 1 h and 30 weeks. The number of animals ranged from 5 to 60 per group. All but one study [73] reported variations in hematologic and/or immunologic parameters upon exposure of the animals to static EF. Direct and indirect mechanisms of the influences of static EF were considered to explain altered hematologic and immunologic parameters. Most of these studies had methodological limitations (see Fig. 4, e.g., allocation of animals to study groups not concealed, no verification of static EF strength, missing control for possible confounders) and it was often not clear from the interpretation of the data what significance they might have for health, i.e., whether static EF have beneficial or detrimental effects on the investigated hematological and immunological parameters in animals.

Möse et al. [72] reported an increased immune response in mice under static EF exposure (static EF strengths between 0.04 and 24 kV/m for 15 days), whereas the immune response was decreased in animals kept in a Faraday cage (zero field). The authors cited these results in support of their hypothesis that exposure to static EF was beneficial and shielding animals from static EF had a negative impact [60].

In a long-term experiment, in which mice were continuously exposed to static EF (2 kV/m) during a period of two years, Kellogg and co-workers found increased values in serum glucose and decreased urea nitrogen levels [41–43]. Furthermore, the mice exposed to the static EF alone lived longest. The authors saw a connection between serum glucose level and lifespan which lent support to their hypothesis that bioelectric processes are involved in mortality and aging rate.

Other studies also consistently reported variations in some blood parameters in rodents upon exposure to static EF. The investigated parameters varied considerably and regarded the serum concentration of various proteins such as albumins and globulins [69, 78], content of hemoglobin and lymphocyte number [50], indicators of oxidative stress [70, 71], Ca^2+^-dependent enzyme activities in the membranes of erythrocytes and mitochondria [74], serum lysozyme activity [75], changes in the surface charge of erythrocytes [77] and number of erythrocytes [76].

Possible mechanisms for the observed alterations of blood parameters were discussed by several authors. Fam [50] discussed his findings in terms of indirect effects of static EF. Living systems are well shielded from the direct influence of EF but the field can act on the skin and fur and thus provide sensory stimulation. Any such interactions may then be transmitted through the blood or the nervous system to deeper body layers. Yet, there was no concrete evidence for this hypothesis in this study. Changes in functional states of enzyme activities [74] and modifications of the surface charge of erythrocytes [77] were discussed to be induced by influences of the static EF on the cell membrane, such as polarization or conformational changes of membrane proteins as well as the modification of the distribution of electric charges. Whether this impact is direct or indirect (for example, via a metabolic cascade) is put up for discussion by the authors [77].

#### Reproduction and development

Three studies examined the reproduction and development of mammals under the influence of a static EF [44, 50, 52]. A replication of the results has not been reported by now. The animals were exposed to static EF between 5.6 kV/m and 340 kV/m and for durations between 4 and 30 months. The size of the study population was between 12 and 50 animals per group.

The data from two extensive laboratory studies on mice were not consistent. Möse and Fischer [52] reported fewer litters in the exposed groups with increasing exposure duration (static EF of 23.8 kV/m for at least 15 days up to 4 month). They did not provide an explanation for this finding because they could not exclude the possibility that this result was raised by chance; the authors therefore suggest that the effect should be verified in upcoming studies. The data on reproduction and development contrasts with the otherwise postulated positive and stimulating effect of the static EF posed by the study authors (see sections Perception mechanism/ Behavioral responses, Histological and biochemical organ parameters and Hematology and immunology). Fam [50], however, did not find an effect of static EF (340 kV/m for 30 weeks) on the number of progenies.

The extensive experimental field study by Angell et al. [44] (observation period of 30 months) provided no evidence for an effect of a HVDC transmission line (mean static EF strength of 5.6 kV/m) on the reproduction and development of cattle (e.g., pregnancy rate, weaning weight) in comparison to a herd kept away from the power line.

#### Genotoxicity

The two studies on genotoxicity - conducted by the same research group - implanted Ehrlich ascites tumor cells in mice [69, 79]. They found chromosomal abnormalities in these tumor cells after a 14-day static EF exposure (8–16 kV/m). Prolongation of exposure and observation period in the second study showed that the effects in exposed mice were transient and disappeared with continued exposure (15 weeks) [79], but these effects have not been confirmed in independent replication studies. The authors assumed that the cells with chromosomal abnormalities died and that only those cells with intact chromosomes survived and proliferated. They further noted that the energy from the applied static EF would have been too low to cause direct effects on biological systems (i.e., a cell-field interaction); thus, the observed effect had to be transmitted via a as of yet unexplained kind of “information”.

#### Therapeutic approaches

The study by Gray et al. [37] points to an improved effect of a chemotherapeutic agent in mice, when it is combined with exposure to static EF (450 kV/m, 4 h/day for 13 days). A significantly greater tumor regression of an implanted mammary adenocarcinoma was observed in the group exposed to the static EF and the chemotherapeutic agent compared to mice that received only the chemotherapeutic agent. This effect has not been replicated as yet. The authors speculated on possibilities how static EF may act on cell functions inside the body: Both the inhomogeneous electrical conductor characteristics of the body and the continuous field variations in and around cells due to its dynamic functioning could entail that static EF are not entirely attenuated (i.e., drop to zero) when reaching the body surface.

## Discussion

### Summary of evidence

The aim of this systematic review was to collect, analyze and evaluate studies addressing effects of static EF on biological functions in humans and vertebrates. Altogether, 48 studies which met criteria for inclusion were evaluated, of which eight studies were conducted with humans and 40 with animals. The animal studies displayed a great degree of heterogeneity with regard to the endpoints and animal species examined, size of study population, the applied EF strengths, and the exposure duration.

A number of studies found evidence that both humans [29, 31, 34] and animals [47–52, 58] are capable of detecting and responding to static EF stimulation. It was suggested that hair movements caused by electrostatic forces play an important role in the perception of static EF fields [34, 48]. Field perception experiments in humans found that detection thresholds for static EF were significantly lower when the whole body was exposed [29, 31] compared to when only the subject’s arm was exposed (partial body exposure) [34]. The most parsimonious reason for this difference is that whole-body exposure in upright posture increases the field strength at the top of the body (head/shoulders) to levels far above that of the nominally applied field, which does not occur with the localized application of the field perpendicular to an outstretched arm. Perception of static EF also appears to be influenced by several other factors such as humidity, awareness, and simultaneous presence of air ions or AC EF. Animal studies further indicated altered behavior upon exposure to static EF, including locomotor activity [49, 51, 52], avoidance behavior [47] and food and water intake [50, 52]. Field perception by humans and animals was replicated by independent investigators.

The vast majority of the evaluated studies dealt with static EF influences on health and physiological functions in humans and animals. An experimental study in visual display unit users found indications that a combination of static EF exposure and high dust concentrations might induce external facial skin irritation [33]. Two other human studies reported that static EF did not induce facial skin symptoms [32] or impair cardiovascular, hematologic, or psychomotor functions [28]. Neither were adverse health effects reported upon long-term exposure to a HVDC power line [35]. A great many of the animal studies reported effects on metabolic activity [49, 56, 60, 62, 68], collagen synthesis [59, 63–65], bone density [61], expression of oxidative stress markers [66, 67, 70, 71, 76], hematologic and immunologic blood parameters [41–43, 50, 69–72, 74–78], neurotransmitter concentrations [56], brain activity [58], litter number [52], genotoxicity [69, 79], and tumor regression [37]. However, the results regarding these parameters were not always consistent and partially contradictory. Some studies could not confirm static EF influences on metabolic functions [52], histological appearance of diverse organ systems [43, 50, 69], neurotransmitter concentrations in the brain [38, 40, 57], functions of the immune system [73] or reproductive and developmental parameters [44].

### Limitations

It is possible that the inconsistencies in the results of the included studies are due to differences in study designs, in particular with regard to the applied EF strengths and variable exposure durations. It should also be noted that the internal validity of the included studies varied considerably and that many studies had elements judged to be susceptible to high risk of bias. The design, conduct and analysis of half of the human studies were largely free of bias, while some sources of bias were identified in the remaining human studies. However, only 22.5% of all animal studies were fully credible in terms of study design and conduct, while 20% of the included studies were susceptible to high risk of bias for most of the rated criteria. Especially in some older studies, the documentation for methods and results did not adhere to practices that now are more common. Consequently, it was not always possible to assess the extent to which confounding factors (like the presence of ozone, air ions, or noise), non-performed measurement and verification of the actual static EF strength, the lack of blinding of the experimenter as to the exposure status and the use of a method with a non-random component to allocate participants/animals to study groups using may have lowered the certainty in the reported exposure effects.

Another potential limitation of this review is that some few studies may possibly have been missed by our search strategy or could not be identified because relevant key words were not found in the title or abstract. Furthermore, our inclusion criteria allowed only articles written in English or German for which a peer-review status was confirmed or could not definitely be excluded. Potentially relevant data published in gray literature or in other languages are therefore not included in this review. Finally, the inclusion of publications with low quality may also have biased the conclusions of this review. Nonetheless, this review represents the most comprehensive summary of the effects of static EF on humans and animals and includes an assessment of the weight of evidence and consistency from individual studies.

## Conclusion

The conclusions of this review are consistent with those of former assessments done for the UK NRPB [8], the Oak Ridge National Laboratory [9], and the WHO [14] that the data, while limited in scope, did not suggest any adverse biological effects of static EF. The strength of this review is that it evaluated more recent studies, a larger number of studies (*n* = 48) than those considered by the WHO (*n* = 7) or the NRPB (*n* = 11), and formally assessed the risk of bias in these studies. The WHO came to the conclusion that further research on the effects of static EF would bring little benefit because the evaluated studies suggested no untoward health effects except for possible stress from prolonged exposure to micro-shocks [14]. In contrast, SCENIHR recommended the collection of data on thresholds for perception, annoyance, and other effects, especially in the presence of varying ion concentrations in the air. These goals are aimed at better defining the likelihood of subjective annoyance from exposure in the vicinity of HVDC power lines [15]. The SSK has recommended performing research projects on human perception of static EF under well-controlled conditions [11]. In light of the currently available data, it is possible that EF strengths underneath high voltage power lines under some conditions are sufficiently high to be detected by humans and animals. The results of this review therefore support the recommendations of SCENIHR and SSK that further research is needed to better define thresholds for field detection. The authors of many studies included in this review furthermore suggested or hypothesized that static EF influences are not restricted to the body surface, but that the fields may also act on physiological functions. Given that no convincing evidence has been provided thus far for primary direct effects of static EF on physiological functions and due to the physical attenuation of static EF at the body surface, a straightforward interpretation of the reported effects and hypotheses on parameters such as metabolic activity, blood parameters, protein synthesis or genetic information e.g., [37, 50, 56, 63, 64, 68, 74, 77] is that these physiological responses occur in response to sensory stimulation of the skin and hair by the EF or were caused by concomitant phenomena of the electrostatic environment such as ozone, air ions or corona that were not appropriately controlled during exposures. In view of the large number of included studies in this review which suffered from severe methodological flaws, we encourage researchers in future studies to achieve a well-controlled and accurate exposure setting when designing experiments involving exposures to static EF. Confidence in the exposure can be achieved through measurements or simulations of the EF strength, meticulous control for possible confounders and unplanned exposure from fields that are not related to the actual exposure, and a randomized, double-blind experimental protocol. Detailed guidance on the complete characterization of EMF exposure has been provided by Valberg [80] and should be considered by any researcher working in EMF exposure science to facilitate the assessment of the comparability of exposures among studies and synthesis of the results.

## Additional file

Additional file 1: Supplemental material including the link for repeating the literature search and a table with the description and five example classifications for the placement of individual human and animal studies into the OHAT 3-tier study quality system. (PDF 139 kb)

## Abbreviations

AC: Alternating current
DC: Direct current
EEG: Electroencephalogram
EF: Electric field
EMF: Electro-magnetic field
GABA: Gamma-aminobutyric acid
HVDC: High-voltage direct current
IARC: International Agency for Research on Cancer
ICNIRP: International Commission on Non-Ionizing Radiation Protection
IEEE: Institute of Electrical and Electronics Engineers
kV/m: Kilovolt/meter
nA/m^2^: Nanoampere/square meter
NRPB: National Radiological Protection Board
OHAT: Office of Health Assessment and Translation
SCENIHR: Scientific Committee on Emerging and Newly Identified Health Risks
SSK: German Commission on Radiological Protection
V/m: Volt/meter
WHO: World Health Organization
