# Supplementary material for: Biological effects of exposure to static electric fields in humans and vertebrates: a systematic review
Source: Environ Health. 2017 Apr 17;16:41. doi: 10.1186/s12940-017-0248-y (PMC5393013; doi:10.1186/s12940-017-0248-y)
Supplement: Additional file 1: — Supplemental material including the link for repeating the literature search and a table with the description and five example classifications for the placement of individual human and animal studies into the OHAT 3-tier study quality system. (PDF 139 kb) [file 12940_2017_248_MOESM1_ESM.pdf]

ADDITIONAL MATERIAL

Biological effects of exposure to static EF in humans and vertebrates: A systematic review

Anne-Kathrin Petri, Kristina Schmiedchen, Dominik Stunder, Dagmar Dechent, Thomas Kraus, William H. Bailey, Sarah Driessen

Literature search strategy

The literature search can be repeated using the following link:

https://www.emf-portal.org/en/article/search/results?keywords=%22static+electric+field%22&logicalOperator=0&authors=&journals=&years=&topics%5B%5D=0&topics%5B%5D=1&topics%5B%5D=2&topics%5B%5D=3&topics%5B%5D=4&topics%5B%5D=5&topics%5B%5D=6&topics%5B%5D=7&topics%5B%5D=8&topics%5B%5D=9&frequencyRanges%5B%5D=0&frequencyRanges%5B%5D=1&frequencyRanges%5B%5D=2&frequencyRanges%5B%5D=3&frequencyRanges%5B%5D=4&timeSpan=0

Table S1: Schematic overview for placement of individual human and animal studies in study quality categories (1st tier, 2nd tier, 3rd tier). Adapted from the approach recommended by the National Toxicology Program's Office of Health Assessment and Translation (2015)[1]. "++" definitely low risk of bias, "+" probably low risk of bias, "-" probably high risk of bias, "--" definitely high risk of bias

			Risk of bias criteria									
			**Key criteria**			Other criteria						
Category	Guidance		**Control for confounding variables**	**Confidence in the exposure**	**Confidence outcome assessment**	Randomization exposure level	Allocation concealment	Identical experimental conditions	Blinding	Attrition/exclusion rate	All outcomes reported	Other potential threats
1st tier	"definitely low" or "probably low" risk of bias for all key risk of bias criteria AND "definitely low" or "probably low" risk of bias for > 50% of all other criteria	Example 1	++	++	+	+	-	++	-	++	+	+
2nd tier	study does not meet criteria for tier 1 or tier 3	Example 2	++	-	++	+	-	+	-	+	++	-
		Example 3	+	-	-	-	-	++	++	--	+	+
		Example 4	-	-	-	+	+	++	-	+	-	+
3rd tier	"probably high" or "definitely high" risk of bias for all key risk of bias criteria AND "probably high" or "definitely high" risk of bias for > 50% of all other criteria	Example 5	--	-	-	-	+	--	-	+	-	-

[1] National Toxicology Program. Handbook for Conducting a Literature-Based Health Assessment Using OHAT Approach for Systematic Review and Evidence Integration. Office of Health Assessment and Translation; 2015. http://ntp.niehs.nih.gov/go/38673
